# Regulation of Amyloid Precursor Protein Processing by the Beclin 1 Complex

**DOI:** 10.1371/journal.pone.0011102

**Published:** 2010-06-15

**Authors:** Philipp A. Jaeger, Fiona Pickford, Chung-Huan Sun, Kurt M. Lucin, Eliezer Masliah, Tony Wyss-Coray

**Affiliations:** 1 Department of Neurology and Neurological Sciences, Stanford University School of Medicine, Stanford, California, United States of America; 2 Institut für Chemie und Biochemie, Freie Universität Berlin, Berlin, Germany; 3 Department of Neurosciences and Pathology, University of California San Diego, La Jolla, California, United States of America; 4 Geriatric Research Education and Clinical Center, Veterans Affairs Palo Alto Health Care System, Palo Alto, California, United States of America; University of Pittsburgh, United States of America

## Abstract

Autophagy is an intracellular degradation pathway that functions in protein and organelle turnover in response to starvation and cellular stress. Autophagy is initiated by the formation of a complex containing Beclin 1 (BECN1) and its binding partner Phosphoinositide-3-kinase, class 3 (PIK3C3). Recently, BECN1 deficiency was shown to enhance the pathology of a mouse model of Alzheimer Disease (AD). However, the mechanism by which BECN1 or autophagy mediate these effects are unknown. Here, we report that the levels of Amyloid precursor protein (APP) and its metabolites can be reduced through autophagy activation, indicating that they are a substrate for autophagy. Furthermore, we find that knockdown of *Becn1* in cell culture increases the levels of APP and its metabolites. Accumulation of APP and APP C-terminal fragments (APP-CTF) are accompanied by impaired autophagosomal clearance. Pharmacological inhibition of autophagosomal-lysosomal degradation causes a comparable accumulation of APP and APP-metabolites in autophagosomes. *Becn1* reduction in cell culture leads to lower levels of its binding partner Pik3c3 and increased presence of Microtubule-associated protein 1, light chain 3 (LC3). Overexpression of *Becn1*, on the other hand, reduces cellular APP levels. In line with these observations, we detected less BECN1 and PIK3C3 but more LC3 protein in brains of AD patients. We conclude that BECN1 regulates APP processing and turnover. BECN1 is involved in autophagy initiation and autophagosome clearance. Accordingly, BECN1 deficiency disrupts cellular autophagy and autophagosomal-lysosomal degradation and alters APP metabolism. Together, our findings suggest that autophagy and the BECN1-PIK3C3 complex regulate APP processing and play an important role in AD pathology.

## Introduction

Alzheimer Disease (AD) affects a growing number of the elderly and results in dramatic loss of cognitive function. It is characterized pathologically by the presence of extracellular beta amyloid (Aβ) assemblies called plaques [1], [2], and intracellular accumulation of Aβ [3] and tau [4]. These lesions are hallmarks of the disease and are associated with neurodegeneration and inflammation [5]. Currently it is unclear how these lesions form, and how protein aggregation and neuronal loss are connected [6]. While much research has centered on abnormal proteolytic processing of Amyloid precursor protein (APP) and tau, less focus has been placed on the possibility that slow, progressive dysfunction of intracellular protein sorting and degradation pathways, such as macroautophagy, may drive pathogenesis steadily over time, especially in cases of sporadic AD [7], [8].

APP is a type I transmembrane protein that can be processed by one of two mutually exclusive cleavage pathways: α-secretase (non-amyloidogenic processing) or β-secretase (amyloidogenic processing) followed by γ-secretase cleavage. Non-amyloidogenic cleavage occurs mainly at the cell surface, whereas amyloidogenic processing takes place in intracellular compartments, including endosomes, lysosomes, and autophagosomes [9], [10]. Amyloidogenic processing releases Aβ which can subsequently be secreted from cells. In addition, APP C-terminal fragments (APP-CTF) of both cleavage pathways can translocate to the nucleus and induce nuclear signaling [11], [12], [13], [14]. Both, Aβ and APP-CTF potentially contribute to AD pathology and can exhibit neurotoxic properties through multiple pathways [15], [16].

APP levels, Aβ levels, and neurodegeneration are tightly coupled. Less than 1% of all AD cases are autosomal dominant early-onset familial AD (FAD) and are caused by mutations in one of three major genes APP, Presenilin-1 (PSEN1), or Presenilin-2 (PSEN2) [17]. These mutations lead to the predominant amyloidogenic cleavage of APP. Additionally, FAD can be caused by APP locus duplication [18] and polymorphisms in the APP promoter region that increase APP levels have been linked to an increased risk for AD [19]. In Down Syndrome an additional copy of chromosome 21, which harbors the *APP* gene, leads to overexpression of APP protein, elevated Aβ levels, plaque deposition and AD-like disease in all older Down's patients [20], [21], [22]. While this illustrates the importance of *APP* gene regulation and APP protein levels in AD, little is known about the regulation of APP metabolism in sporadic AD cases. The levels of APP protein and APP mRNA in AD cases versus control has been reported in the past with conflicting results, but more recent research indicates increased levels of APP and APP-CTFs in sporadic AD brains [23], [24], [25], [26].

Macroautophagy (in this paper referred to as ‘autophagy’) is a major pathway involved in the degradation of long-lived proteins, protein aggregates, and organelles, cellular remodeling, and survival during starvation [27], [28]. Autophagy is characterized by the formation of a cup-shaped isolation membrane that develops around cytosolic components and eventually fuses to form a double membrane bound vesicle [29], [30], [31], [32]. The protein Microtubule-associated protein 1, light chain 3 (LC3) is anchored via conjugated phosphatidylethanolamine to the vesicle's membrane. While the un-conjugated LC3 is called LC3-I, the phosphatidylethanolamine conjugated LC3 is referred to as LC3-II and is a specific marker for these so-called autophagosomes [33]. Autophagosomes then undergo several microtubule- [34] and dynein-dependent maturation events [35], [36], including fusions with multivesicular bodies, early and/or late endosomes [37], before eventually fusing with lysosomes [38], [39].

Autophagy has recently been implicated in a number of diseases including neurodegenerative conditions and it appears that autophagy can exert both a pathological or protective role, depending on the setting [40]. While it is still largely unknown how dysfunction of the autophagy pathway might contribute to neurodegeneration and AD, recent papers suggest a role for Beclin 1 (BECN1) in AD and mild cognitive impairment [41], [42], [43]. Haploinsufficiency of *Becn1* in mice decreases neuronal autophagy and promotes neuronal degeneration [41]. Moreover, in a mouse model for AD genetic reduction of *Becn1* expression results in increased accumulation of APP fragments and Aβ, increased neurodegeneration and increased inflammation [41]. In addition, Autophagy has been shown to protect neurons from Aβ induced cytotoxicity [44], [45], [46].

BECN1 plays an important role in autophagy [47], [48], [49], [50], [51] and is the human homolog of the yeast autophagy protein Atg6/Apg6 [52]. BECN1 forms a core complex with the class 3 phosphoinositide-3-kinase PIK3C3 (also known as Vps34) [51], [53], [54]. Other proteins such as UVRAG, Atg14L, PIK3R4/Vps15, Ambra1, Rubicon, or Bif-1, join this complex depending on its physiological function in autophagy or endosomal trafficking [55], [56], [57]. *Becn1* and *Pik3c3* mRNA and protein are expressed in human and mouse brains [40] (Fig. S1, from the Allen Mouse Brain Atlas. Seattle (WA): Allen Institute for Brain Science. Available from: http://mouse.brain-map.org [58]). Knockout mice lacking *Becn1* (*Becn1^−/−^*) die during embryogenesis [48], [50].

To date, the mechanism describing how deficiency in BECN1 can cause changes in APP processing and amyloid accumulation are unknown. Here we characterize the relationship between BECN1 levels, autophagy, and APP processing in cell culture and in human brain tissue. We show that intracellular APP, APP-CTFs, and Aβ can be reduced by autophagy activation and that the BECN1-PIK3C3 complex regulates APP processing and accumulation.

## Results

### Activation of autophagy promotes APP, APP-CTF, and Aβ degradation

**Figure 1:**
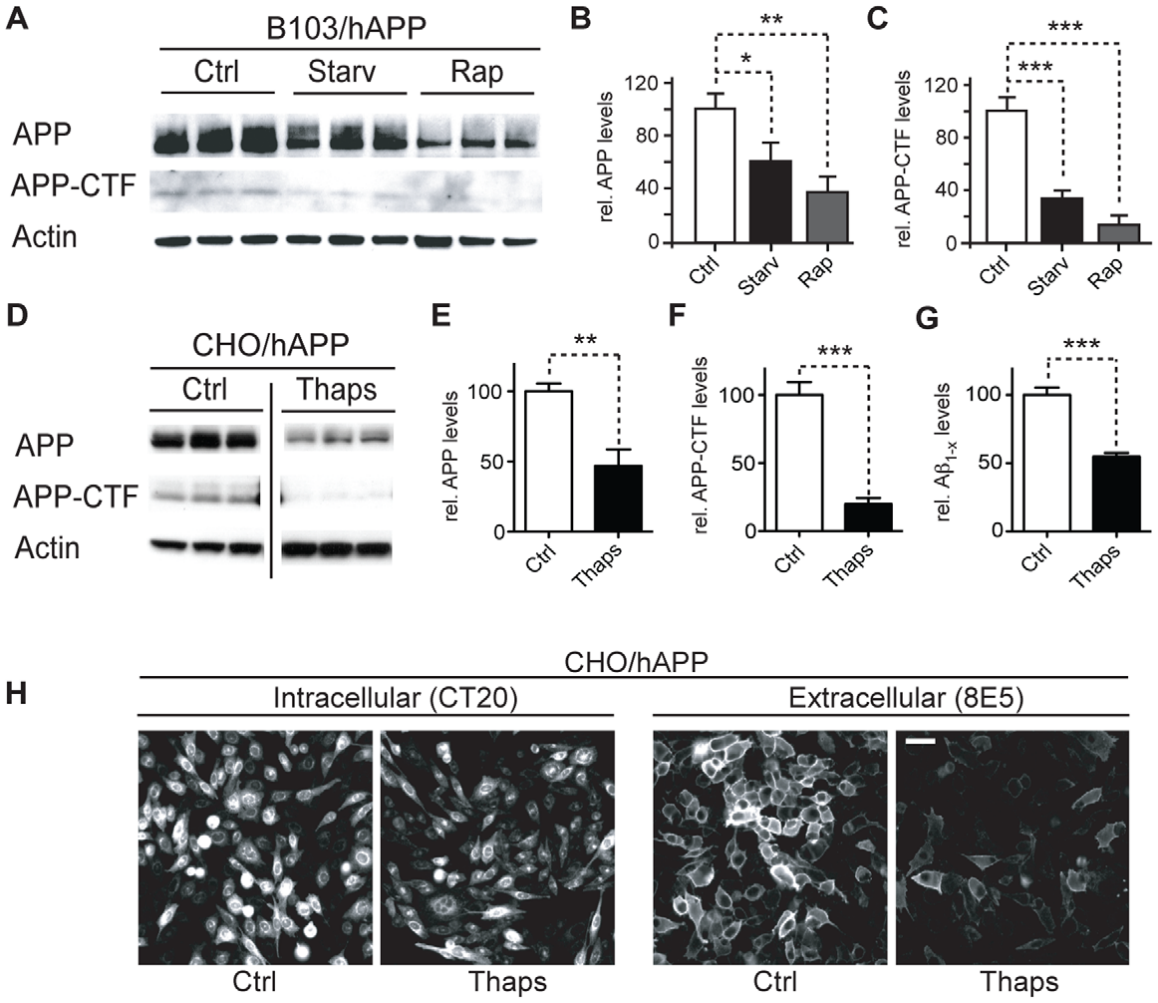
Activation of autophagy promotes APP, APP-CTF, and Aβ degradation. **A–C.** B103/hAPP cells were left untreated (Ctrl), starved for 90 min in HANKS solution (Starv), or treated with 100 nM rapamycin in DMEM (Rap) for 90 min. Western blots (A) and quantification (B, C) of RIPA cell lysates probed with the CT15 antibody recognizing full-length APP and APP-CTFs and with an actin antibody as a control for loading. **D–F.** CHO/hAPP cells were left untreated (Ctrl) or treated for 12 hrs with 3 µM thapsigargin (Thaps) in DMEM/10%FBS. Western blots (D) and quantification (E, F) of RIPA cell lysates probed with antibodies as in A. (Data from the same blot. The vertical line indicates removal of three lanes not part of this experiment.) **G.** Secretion of Aβ into the cell supernatant was measured by ELISA (12 hrs/1 µM Thaps) **H.** Epifluorescence microscopy images of CHO/hAPP cells treated as in D, permeabilized with Tween and stained with antibody CT20 to label all cellular APP, or not permeabilized and stained with antibody 8E5 which recognizes the ectodomain of APP at the cell surface (scale bar represents 25 µm). Bars are mean ± SEM from triplicate cultures of at least two independent experiments. * p<0.05, ** p<0.01, *** p<0.001 by unpaired Student's t test.

Activation of autophagy can lead to degradation of α-synuclein, huntingtin, and poly-ubiquitinated proteins [59], [60], [61], [62]. To test whether APP and APP-CTFs can also be reduced through this mechanism, we induced autophagy in B103 rat neuroblastoma cells which lack endogenous rat APP and are stably transfected with wildtype human APP695 (B103/hAPP) [63]. To induce autophagy we used either starvation [64] or rapamycin treatment which inhibits mTOR and activates autophagy [65] (Fig. 1A). APP and APP-CTF levels were significantly reduced in starved B103/hAPP cells and further reduced in rapamycin treated B103/hAPP cells (Figure 1B–C). Rapamycin treatment did not affect APP mRNA levels analyzed by qRT-PCR (data not shown). Furthermore, inhibition of autophagy through lenti-viral Atg5 siRNA significantly impaired starvation-induced autophagosomal clearance of APP (Fig. S2). Similar to the findings in neuronal cells, Chinese hamster ovary (CHO) cells stably transfected with human APP695 (CHO/hAPP) [12] and treated with the autophagy inducer thapsigargin [66] showed a more than 50% reduction in APP and APP-CTF levels (Fig. 1D–F) and significantly reduced levels of Aβ in the cell supernatant (Fig. 1G). Consistent with these biochemical findings, microscopy (Fig. 1H) revealed reductions both in intracellular APP (detected with CT20 antibody) and in cell surface APP (detected on non-permeabilized cells with 8E5 antibody). These findings indicate that autophagy activation can reduce levels of APP and APP metabolites.

### *Becn1* knockdown increases APP, APP-like proteins, APP-CTFs and Aβ

**Figure 2:**
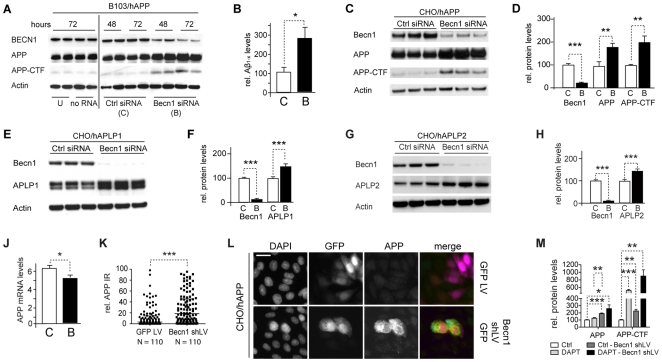
*Becn1* knockdown increases APP, APP-like proteins, APP-CTFs and Aβ. **A–B.** B103/hAPP cells were treated with *Becn1* siRNA for 48–72 hrs. Cells were left untreated (U), treated with transfection reagent alone (no RNA), treated with scrambled siRNA (Ctrl siRNA [C]), or treated with Becn1 siRNA (Becn1 siRNA [B]). Western blots (A) of RIPA cell lysates were probed with a Becn1 antibody, the CT15 APP antibody, and with an actin antibody as a control for loading. For quantification see Fig, S2. (Data from two blots with identical exposure times. Blot border indicated by vertical black line.) Total Aβ_1-x_ concentrations measured by ELISA in cell culture supernatant from the same cells at 72 hrs (B). **C–D.** CHO/hAPP cells were treated with *Becn1* siRNA for 48 hrs. Western blots (C) and quantification (D) of RIPA cell lysates that were probed with a Becn1 antibody, the CT15 APP antibody, and with an actin antibody as a control for loading. **E–F.** CHO/APLP1 cells were treated with *Becn1* siRNA for 48 hrs. Western blots (E) and quantification (F) of RIPA cell lysates that were probed with a Becn1 antibody, an APLP1 antibody, and with an actin antibody as a control for loading. **G–H.** CHO/APLP2 cells were treated with *Becn1* siRNA for 48 hrs. Western blots (G) and quantification (H) of RIPA cell lysates that were probed with a Becn1 antibody, an APLP2 antibody, and with an actin antibody as a control for loading. **J.** Levels of APP mRNA were compared by qRT-PCR in scrambled [C] or *Becn1* [B] siRNA treated B103/hAPP cells. **K–L.** CHO/hAPP cells were treated with either GFP lentivirus or *Becn1* shRNA-GFP lentivirus. Quantification of the relative APP immunofluorescence (K) and epifluorescence microscopy (L) of GFP lentivirus or *Becn1* shRNA-GFP lentivirus treated permeabilized CHO/hAPP cells, probed with DAPI and CT20 APP antibody (scale bar represents 10 µm). **M.** Inhibition of γ-secretase activity through 100 nM DAPT treatment had no significant effect on APP levels and an additive effect on APP-CTF accumulation with *Becn1* shLV treatment. Bars are mean ± SEM from duplicate/triplicate cultures of at least two independent experiments. * p<0.05, ** p<0.01, *** p<0.001 by unpaired Student's t test.

The reported reduction in BECN1 in AD brains [41], [42] and the increased plaque formation and neurodegeneration in *Becn1* ^+/−^ *APP* mice [41] led us to investigate whether Becn1 deficiency affected APP production, processing, or degradation *in vitro*. Reduction of *Becn1* by siRNA in B103/hAPP cells more than doubled the levels of cellular APP and APP-CTFs (Fig. 2A and S3). Moreover, the reduced levels of Becn1 also increased the amount of secreted Aβ in the cell culture supernatant when compared to cells treated with a scrambled control siRNA (Fig. 2B). Similar results were obtained with two different siRNA sequences (data not shown). CHO/hAPP cells treated with *Becn1* siRNA also showed twofold increases in APP and APP-CTFs (Fig. 2 C and D). This prominent increase in APP protein in *Becn1* siRNA treated cells could also be visualized and quantified with fluorescent microscopy showing increased immunoreactivity for both, C-terminal (CT20) and N-terminal (8E5) APP antibody stains (Fig. S4).

Reduced autophagic activity could be specific for APP degradation or it could also affect the processing of amyloid precursor-like proteins. Both, Amyloid precursor like protein-1 (APLP1) and Amyloid precursor like protein-2 (APLP2) are substrates of α-, γ-, and ε-secretase cleavage in a similar manner as APP, while APLP2 can also be cleaved by β-secretase [67]. APP, APLP1, and APLP2 can form homo- and heterodimers [68], making it possible that they are affected similarly by processing alterations. To test if autophagy plays a role in APLP1 and APLP2 degradation, we applied *Becn1* siRNA to cell lines stably expressing human APLP1 or APLP2 [12]. Reducing Becn1 in CHO/hAPLP1 and CHO/hAPLP2 cells resulted in significant increases in APLP1 (Fig. 2E–F) and APLP2 levels, respectively (Fig. 2G–H).

To exclude the possibility that the observed cellular changes in APP, APP-CTF, and Aβ levels in response to *Becn1* siRNA could be accounted for by transcriptional up-regulation of APP mRNA levels, we performed qRT-PCR on *Becn1* or control siRNA treated B103/hAPP cells. APP mRNA levels decreased slightly in *Becn1* siRNA treated B103/APP cells (Fig. 2J), therefore increases in APP, APP-CTFs, and Aβ cannot be attributed to increased transcription of the precursor.

To reduce potential transfection related effects, we transduced CHO/hAPP cells with a lentivirus (LV) containing either a control plasmid encoding only GFP (GFP LV) or a GFP plasmid encoding a *Becn1* shRNA sequence (*Becn1* shLV; different sequence from the siRNA's used above). The *Becn1* shRNA LV treated cells exhibited a significant increase in APP immunofluorescence when compared to GFP LV treated control cells (Fig. 2K–L).

In the *Becn1* siRNA treated cells there was a significant correlation between Aβ and APP, and between Aβ and APP-CTFs (R = 0.619, p = 0.03 and R = 0.698, p = 0.01, respectively, data not shown), suggesting that the increase in secreted Aβ was due to increased levels of its precursor, APP. The Aβ/APP ratio was similar in control and *Becn1* siRNA treated B103/APP cells (data not shown), suggesting unchanged γ-secretase activity. To further test the role of γ-secretase in the observed effects, we treated control or *Becn1* shLV transduced B103/hAPP cells with DAPT, a γ-secretase inhibitor. This treatment had no significant effect on the accumulation of full-length APP in control cells (Fig. 2M and S5) and did not significantly enhance the levels of full-length APP in *Becn1* shLV treated cells any further. The APP-CTF levels on the other hand were significantly increased after DAPT treatment (indicating successful γ-secretase inhibition) and this effect was additive when DAPT was applied together with *Becn1* shLV. These results indicate that the accumulation of APP and APP-CTFs in the Becn1 deficient cells are unlikely the result of substantial changes in γ-secretase activity.

In summary, these findings show that reduced Becn1 levels can cause intracellular accumulation of APP and its metabolites and increased secretion of Aβ. This accumulation appears not to be restricted to APP but also affects other APP-family members, suggesting that the observed accumulations are due to changes in shared processing and trafficking pathways. Finally, the buildup of APP and APP-CTFs mediated by Becn1 deficiency appears to be independent of γ-secretase activity.

### Overexpression of APP does not change Becn1 or Pik3c3 protein levels

**Figure 3:**
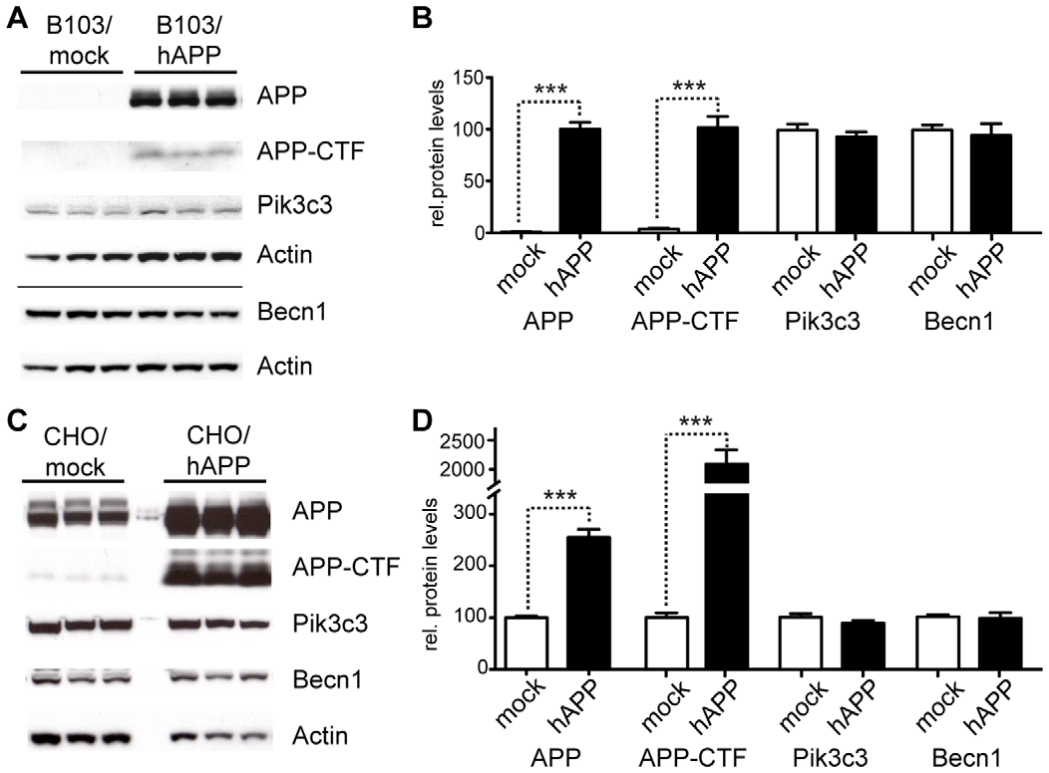
Overexpression of APP does not change Becn1 or Pik3c3 protein levels. **A–B.** B103 cells were stably transfected with an empty plasmid (mock) or a hAPP encoding plasmid. Western blots (A) and quantification (B) of RIPA cell lysates that were probed with the CT15 APP, a Becn1, and a Pik3c3 antibody. An actin antibody was used as a loading control. **C–D.** CHO cells were stably transfected with an empty plasmid (mock) or a hAPP encoding plasmid. Western blots (C) and quantification (D) of RIPA cell lysates that were probed with the CT15 APP, a Becn1, and a Pik3c3 antibody. Actin antibody was used as a loading control. Bars are mean ± SEM from triplicate cultures of at least two independent experiments. * p<0.05, ** p<0.01, *** p<0.001 by unpaired Student's t test.

Brains from AD patients contain less BECN1 protein and mRNA than non-demented controls [41], [42], [43]. This reduction could be caused by a disease-related (BECN1-independent) increase in APP levels. To measure the effects of APP expression on Becn1 and Pik3c3 levels, we compared B103 cells that were stably transfected with a mock vector and express no endogenous APP (B103/mock) with cells that were stably transfected with human APP (B103/hAPP; these cells express close to endogenous levels of APP [69]) (Fig. 3A). While APP and APP-CTF levels were strongly increased, Becn1 and Pik3c3 levels were unchanged in B103/hAPP cell compared to B103/mock cells (Fig. 3B).

Expression levels of APP that are chronically much higher than normal could have an effect on Becn1 and Pik3c3 levels. To measure the effects of higher than endogenous levels of APP expression on Becn1 and Pik3c3 levels, we compared CHO cells that were stably transfected with a mock vector and express only endogenous hamster APP (CHO/mock) with cells that were stably transfected with a hAPP vector and express high hAPP levels (CHO/hAPP) (Fig. 3C). Becn1 and Pik3c3 levels remained unchanged despite a strong elevation in APP and APP-CTF levels in these cells (Fig. 3D). These findings indicate that the levels of cellular APP or APP-CTF do not directly influence Becn1 and Pik3c3 levels.

### Reduction of Becn1 impairs degradation of autophagosomes and reduces Pik3c3 levels

**Figure 4:**
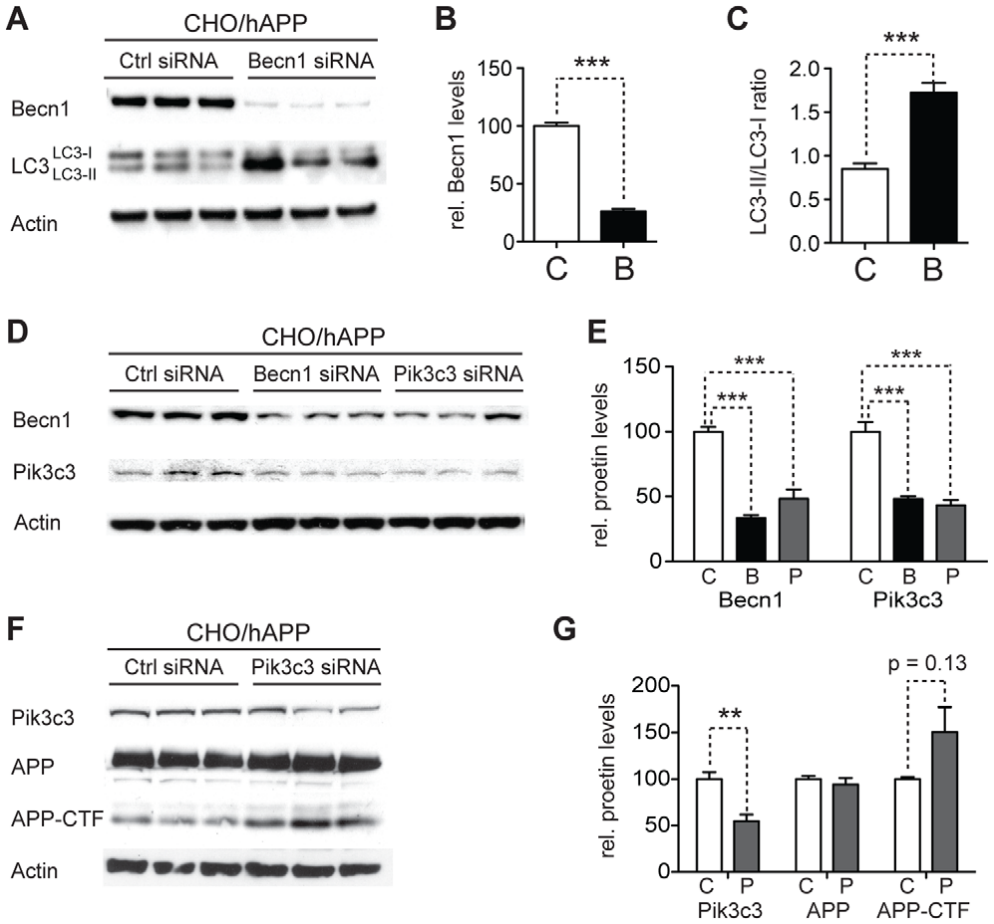
Reduction of Becn1 impairs degradation of autophagosomes and reduces Pik3c3 levels. **A–C.** CHO/hAPP cells were treated with *Becn1* siRNA for 48 h. Western blots (A) of RIPA cell lysates were probed with a Becn1 and LC3 antibody. An actin antibody was used as a loading control. Quantification (B) of the Becn1 band intensity and the ratio of LC3-II to LC3-I (C). **D–E.** CHO/hAPP cells were treated with *Becn1* and *Pik3c3* siRNA for 48 h. Western blots (D) and quantification (E) of RIPA cell lysates that were probed with a Becn1 and Pik3c3 antibody. An actin antibody was used as a loading control. **F–G.** CHO/hAPP cells were treated with *Pik3c3* siRNA for 48 h. Western blots (F) and quantification (G) of RIPA cell lysates that were probed with the CT15 APP antibody and with an actin antibody as a control for loading. Bars are mean ± SEM from triplicate cultures of at least two independent experiments. * p<0.05, ** p<0.01, *** p<0.001 by unpaired Student's t test.

To investigate how the observed effects of Becn1 reduction on APP-family protein processing can be linked to autophagy, we measured the levels of the autophagosomal marker LC3-II in *Becn1* siRNA treated CHO/hAPP, CHO/hAPLP1, and CHO/hAPLP2 cells (Fig. 4A and data not shown). A 75% knockdown of Becn1 (Fig. 4B) caused a significant shift in the LC3-II/LC3-I ratio indicating an accumulation of autophagosomes in all three cell lines (Fig. 4C and data not shown).

Becn1 is a core component of the class 3 PI3 kinase complex [70]. Reduction of Becn1 levels could affect the stability of this complex and influence the levels of other proteins in the complex. To address this possibility we measured the levels of Pik3c3 in response to *Becn1* siRNA treatment, and the levels of Becn1 in response to *Pik3c3* siRNA (Fig. 4D). The cellular levels of both proteins, Becn1 and Pik3c3, appear to be linked, with the reduction of one leading to a comparable reduction of the other (Fig. 4E).

These findings led us to investigate if Pik3c3 reduction by itself can cause a change in APP processing, similar to *Becn1* siRNA (Fig. 4A). While we observed a trend towards increased APP-CTF in *Pik3c3* siRNA treated cells, we found no significant differences (Fig. 4F–G). These data support a central role for Becn1 in modulating APP levels.

### Inhibition of autophagosome turnover leads to a reduction in Becn1 and Pik3c3 levels

BECN1 is reduced in AD brains [41], [42], [43], however the mechanism behind this reduction is unknown. One hypothesis is that impaired autophagosomal-lysosomal function may activate a negative feedback loop that subsequently reduces BECN1 levels. It is conceivable that this homeostatic loop could become activated after autophagy is impaired in order to prevent apoptosis or autophagic cell death [71]. An accumulation of autophagosomes in AD brain tissue (indicating impaired autophagosomal degradation) has been reported previously [7], [10], [72], [73]. To test this hypothesis we inhibited autophagosomal-lysosomal fusion using bafilomycin A1 (BafA) [74], [75]. BafA treatment has been shown to lead to accumulation of APP and APP-CTFs in late endosomes and multivesicular bodies (MVB) [76]. We tested if BafA treatment can also lead to APP and APP-CTF accumulation in autophagosomes and if the accumulation of these autophagosomes has any effects on Becn1 or Pik3c3 levels.

**Figure 5:**
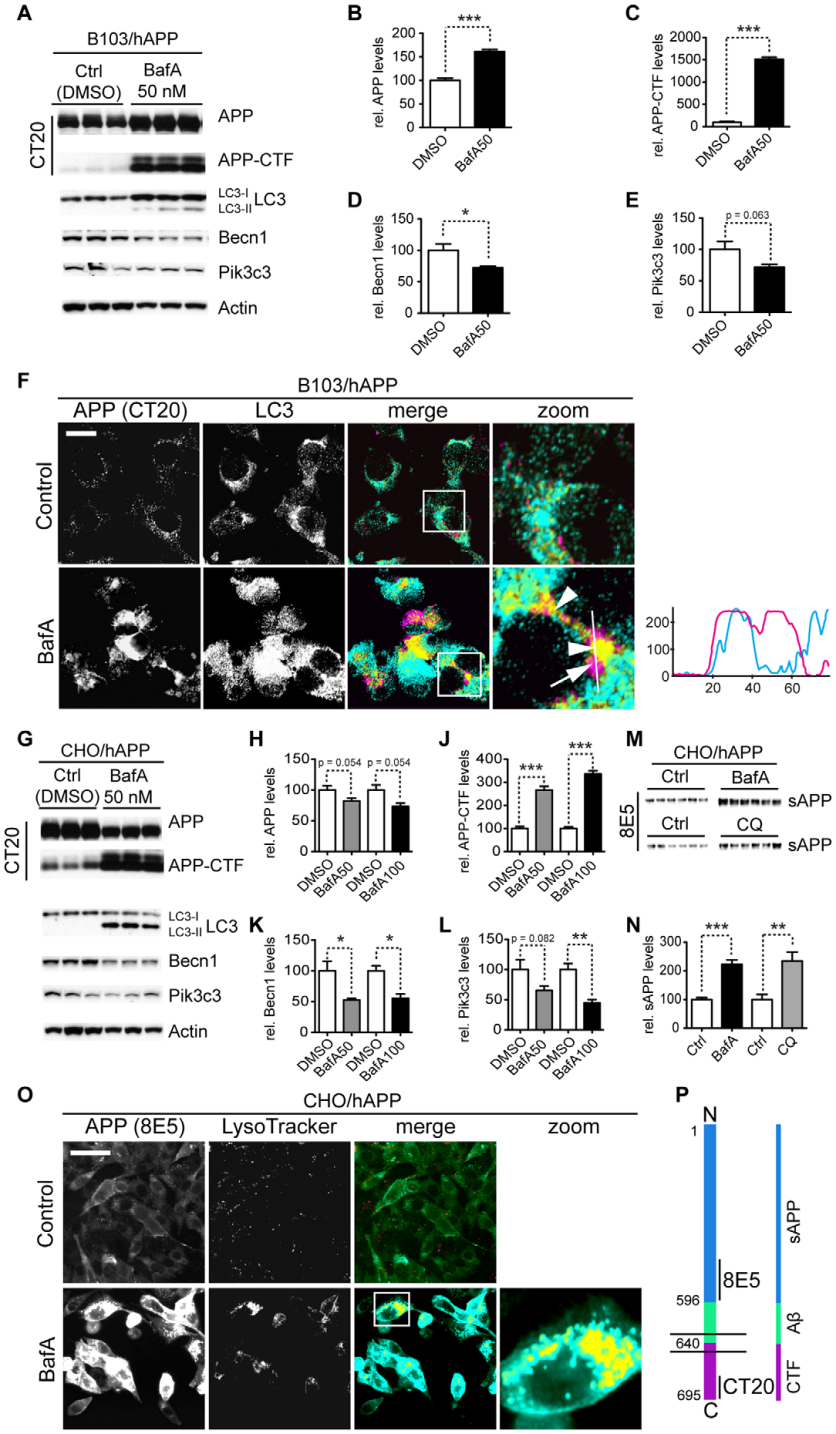
Inhibition of autophagosomal turnover leads to a reduction in Becn1 and Pik3c3 levels. **A–E.** B103/hAPP cells were treated with vehicle (DMSO) or 50 nM BafA for 24 hrs to inhibit autophagosomal degradation. Western blots (A) and quantification (B–E) of RIPA cell lysates that were probed with CT15 APP, LC3, Becn1, and Pik3c3 antibody. An actin antibody was used as a loading control. **F.** Confocal microscopy of B103/hAPP cells treated with vehicle (DMSO) or 100 nM BafA for 24 hrs. Cells were stained with CT20 APP antibody (magenta) and LC3 antibody (cyan). Co-localization is indicated in yellow. Arrowheads indicate LC3 positive APP containing vesicles. The arrow indicates an APP containing LC3 negative vesicle (scale bar represents 10 µm). The line indicates cross-section. Cyan line in the cross-section represents APP intensity, magenta line represents LC3 intensity (AU). **G–L.** CHO/hAPP cells were treated with vehicle (DMSO), 50 nM, or 100 nM BafA (WB data not shown) for 24 hrs. Western blots (G) and quantification (H–L) of RIPA cell lysates that were probed with the CT15 APP, LC3, Becn1, and Pik3c3 antibody. An actin antibody was used as a loading control. **M–N.** BafA and CQ treatment cause increased APP processing which in turn leads to elevated levels of secreted APP (sAPP) in the cell supernatant (M). This is quantified in (N). **O.** Epifluorescence microscopy of CHO/hAPP cells treated with vehicle (DMSO) or 100 nM BafA for 12 hrs. Cells were stained with the 8E5 APP antibody (magenta) and LysoTracker (cyan). Co-localization is indicated in yellow (scale bar represents 25 µm). **P.** Schematic representation of the APP antibody epitopes. Bars are mean ± SEM from triplicate cultures of at least two independent experiments. * p<0.05, ** p<0.01, *** p<0.001 by unpaired Student's t test.

In B103/hAPP cells BafA treatment led to a strong increase in APP and APP-CTFs compared to vehicle treated cells (Fig. 5A–C). It also led to a significant accumulation of LC3-I and LC3-II (Fig. 5A), indicating a successful inhibition of autophagosomal degradation through BafA treatment. This impairment of autophagy caused a significant decrease in Becn1 (Fig. 5D, p = 0.025) and reduced, but not significantly changed, Pik3c3 levels (Fig. 5E, p = 0.063). Microscopy revealed that APP accumulates primarily in large vacuoles in the perinuclear space (Fig. 5F). Some APP containing vesicles stained positive for LC3 (Fig. 5F, arrowheads) but APP also accumulated in large non-LC3 positive vesicles (Fig. 5F, arrow). In vehicle treated cells only very little APP was found in LC3 positive compartments and these compartments were small in size (Fig. 5F).

Similar results were obtained for CHO/hAPP cells, where treatment with BafA also led to a reduction in Becn1 and Pik3c3 protein levels respectively (Fig. 5G, 5K–L). While CT20 full length APP immunoreactivity slightly decreased (Fig. 5H), a strong increase in APP-CTFs (Fig. 5J) and in sAPP (Fig. 5M–N) were observed. The reduction of full-length APP in CHO/hAPP cells (Fig. 5G–H) can be attributed to elevated intracellular and extracellular cleavage of APP. The antibody used in Fig. 5G (CT20) does not recognize the N-terminal cleavage product (Fig. 5P) and enhanced APP processing will lead to an apparent reduction in intracellular (full-length) APP (CT20) levels. Accordingly, the N-terminal sAPP cleavage product accumulates both in the cell supernatant (Fig. 5M–N) and in intracellular, LysoTracker-positive vesicles (Fig. 5O) when probed with the N-terminal antibody 8E5. Total APP and its metabolites accumulate in CHO/hAPP cells, consistent with a disruption in autophagosomal degradation.

To explore alternative inhibitors of autophagosomal-lysosomal degradation and rule out unspecific BafA effects, we compared control, BafA, chloroquine (CQ), and ammonium-chloride/leupeptin (NL) treated CHO/hAPP and B103/hAPP cells (Fig. S6). We found that both CQ and NL cause an accumulation of APP and APP-CTFs, very similar to BafA. This strongly supports the hypothesis that APP is indeed processed through the autophagosomal-lysosomal pathway. Interestingly, we observe the highest reduction in Becn1 and Pik3c3 levels after BafA treatment. CQ treatment causes a slight (p = 0.06) reduction in Becn1 and a significant reduction in Pik3c3, while NL has no significant effect on Becn1 or Pik3c3. BafA inhibits autophagosomal-lysosomal fusion, while the two other treatments primarily inhibit autolysosomal degradation. This supports the hypothesis that the accumulation of autophagosomes, rather then the inhibition of lysosomal degradation, affects Becn1 and Pik3c3 levels in a negative feedback-loop.

We conclude that inhibition of autophagosomal-lysosomal fusion through pharmacological treatments leads to accumulation of APP, APP-CTF, sAPP, and autophagosomes. This accumulation results in a reduction of Becn1 and Pik3c3 levels, possibly through a negative feedback mechanism.

### Becn1 overexpression reduces APP immunoreactivity

**Figure 6:**
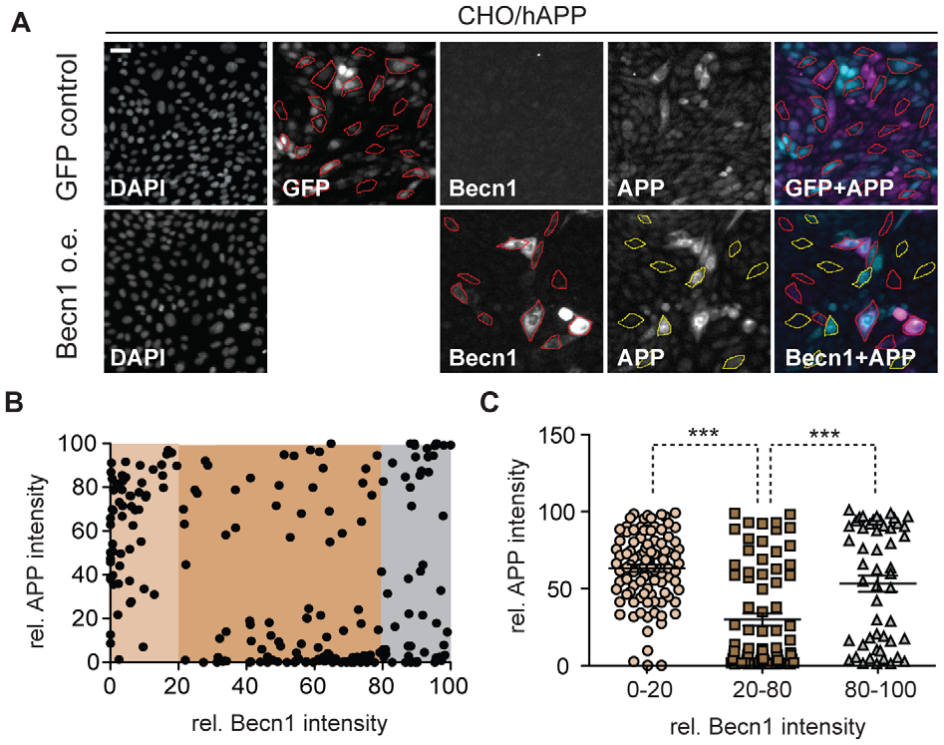
Becn1 overexpression reduces APP immunoreactivity. **A.** CHO/hAPP cells were transduced with either a *GFP* LV (GFP control) or a *mBecn1* LV (Becn1 o.e.). Epifluorescence microscopy was performed after staining with Becn1 and APP CT15 antibodies (Scale bar represents 25 µm). *GFP* LV transduced cells show very faint Becn1 immunoreactivity, while *Becn1* LV transduced cells exhibit a range of Becn1 signal intensity. No GFP signal is present in the *Becn1* LV cells. A random selection of cells (N = 214) was picked from the *GFP* LV cells and the *Becn1* LV cells. The *Becn1* LV cells were randomly selected in both, the APP (yellow outline) and the Becn1 (red outline) channel. **B.** Relative immunofluorescence of the selected cells (AU). They can be divided in low, medium, and high Becn1 expressing cells. **C.** Quantification of the relative APP immunofluorescence in the three cohorts. Medium Becn1 overexpression leads to a significant reduction in APP levels. Medians were compared by Man-Whitney U test. * p<0.05, ** p<0.01, *** p<0.001

To determine if *Becn1* overexpression alone can reduce APP baseline levels we transduced CHO/hAPP cells with either a *Becn1* LV or a control GFP LV (Fig. 6A). While baseline Becn1 levels give only very faint immunoreactivity in fluorescent microscopy, the *Becn1* LV treated cells exhibited a wide range of Becn1 expression levels (from baseline to strong overexpression, Fig. 6A). We randomly selected N = 214 *Becn1* LV treated cells covering the whole spectrum of Becn1 expression from both, the Becn1 (red outline) and APP channel (yellow outline), and measured their relative Becn1 and APP immunofluorescence (Fig. 6B). Next, we grouped these cells into low (<20th percentile), medium (20–80th percentile), and high (>80th percentile) Becn1 expressing cells and compared the median APP immunofluorescence in these groups (Fig. 6C). While no or low overexpression of Becn1 has no effect on APP immunoreactivity (Fig. 6C, 0–20), medium overexpression significantly reduces baseline APP levels (Fig. 6C, 20–80). Very strong, and likely non-physiological overexpression of Becn1 (Fig. 6C, 80–100) had no lowering effect on APP immunoreactivity, but led to either abnormally decreased or increased cell size, indicating that these very high levels of Becn1 expression might impair cellular homeostasis (Fig. S7A–B). This last finding is similar to very high overexpression of GFP protein and probably an artifact. For more details on the effects of GFP overexpression in the control cells, see supplemental Fig. S7 B. These results suggest that moderate increases in Becn1 levels alone can have an APP-lowering effect in CHO/hAPP cells, as long as Becn1 is not expressed at extremely high and probably non-physiological levels.

### AD brains have less BECN1 and PIK3C3 and more LC3

**Figure 7:**
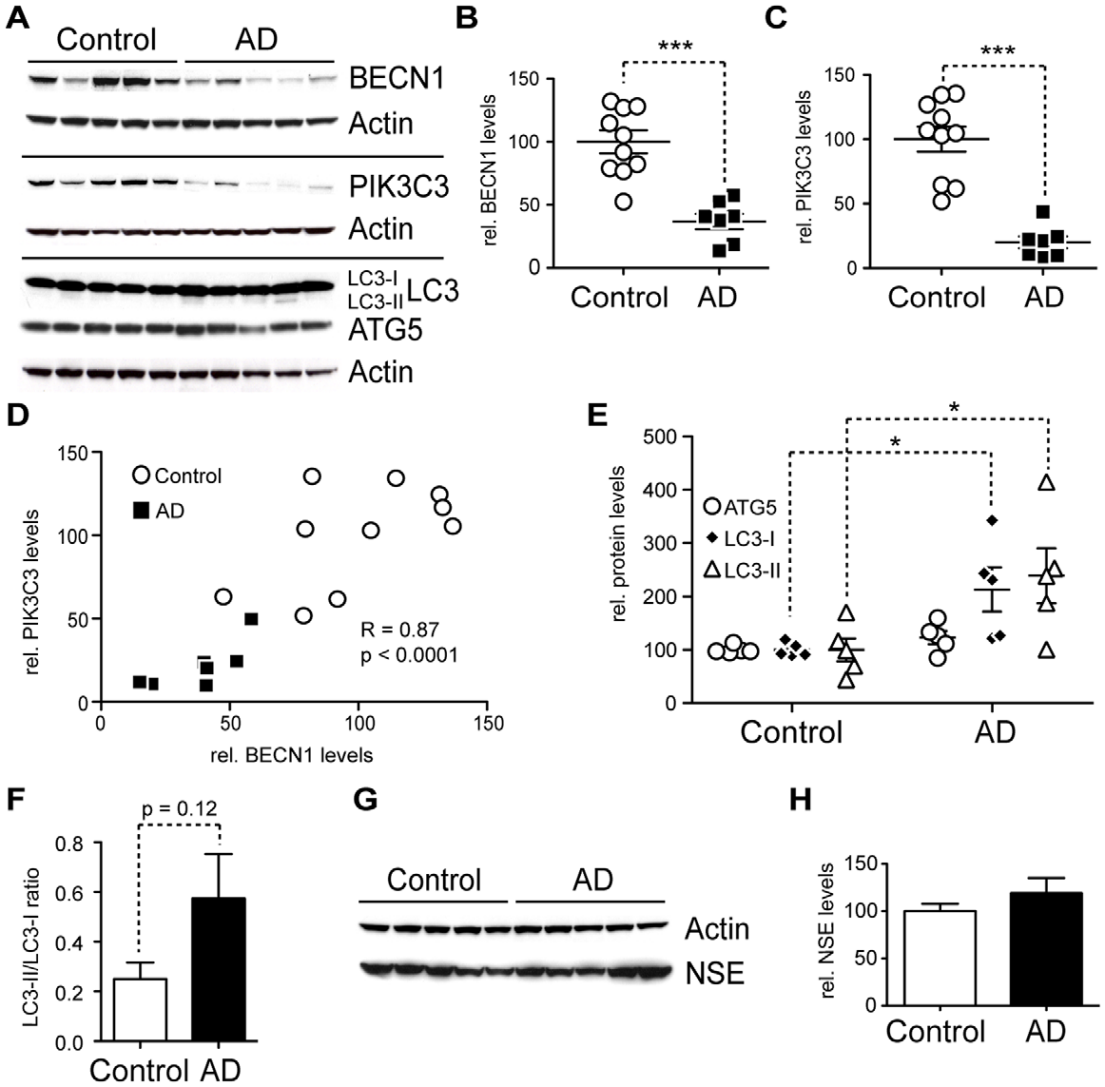
AD brains have less BECN1 and PIK3C3 and more LC3. **A–H.** Comparison of protein levels in frontal cortex (gray matter) from AD brains and age matched, non-demented, non-pathological controls. Western blots (A) and quantification (B–F) of RIPA lysates that were probed with the CT20 APP, LC3, Becn1, Pik3c3, and Atg5 antibody. An actin antibody was used as a loading control. 7 AD and 10 control cases were used. BECN1 and PIK3C3 levels were significantly reduced in AD cases (B–C). A significant linear correlation exists between BECN1 and PIK3C3 levels (R = 0.86, p<0.0001), consistent with them functioning in a complex (D). While ATG5 levels appear unchanged, LC3-I and LC3-II levels are significantly elevated (E). A slight trend was detected in LC3-II/LC3-I ratio change (F). No significant difference could be detected in the levels of a neuronal marker NSE between the control and AD brains, indicating that the observed changes are not due to gross neuronal loss (G and H). All scattergrams show mean ± SEM. Means were compared by unpaired Student's t test. * p<0.05, ** p<0.01, *** p<0.001

BECN1 and PIK3C3 form a complex with PI3 kinase (PI3K) activity that is necessary for the classical autophagy-activating pathway through mTOR. We and others have previously shown that BECN1 is strongly and specifically reduced in affected regions of Alzheimer's disease (AD) brains [41], [42], [43]. Heterozygous deletion of *Becn1* in an AD mouse model caused increased neurodegeneration, decreased autophagy, and disruption of the lysosomal system [41]. Our cell culture findings presented above indicate that BECN1 plays an important role in APP processing and trafficking and that BECN1 reduction has effects on the PI3K complex stability and autophagosomal degradation. To understand if the observed reduction of BECN1 in AD patients is an isolated finding or if it could causes a more general disturbance of the autophagosomal system (similar to our *in vitro* findings) we measured multiple key proteins involved in autophagy (Fig. 7A) in human brain samples. Protein was extracted from cortical gray matter of confirmed Alzheimer disease patients (N = 7, age 81±12.6 years, MMSE 4.3±6.1) and non-demented control subjects (N = 10, age 77.7±8.1 years, MMSE 28.3±3.0), using a detergent containing extraction buffer (RIPA). We found PIK3C3 and, consistent with our previously published findings [41], BECN1 to be strongly reduced in AD brains when compared to non-demented age-matched controls (Fig. 7B–C). There was a highly significant correlation between the amount of BECN1 and PIK3C3 (Fig. 7D, R = 0.86, p<0.0001) in agreement with their combined role in forming the autophagy inducing PI3K complex. In support of previous findings by others [10], we measured elevated levels of LC3-I and LC3-II in AD patient brains (Fig. 7E) and we observed a trend towards higher LC3-II/LC3-I ratios (Fig. 7F). In contrast, expression levels of another autophagy protein, ATG5 were unchanged in AD brains, indicating that only portions of the autophagy pathway are de-regulated in AD (Fig. 7A&E). To ensure that the observed reduction in BECN1 and PIK3C3 levels cannot be attributed to a gross decrease in neuronal mass, we measured the levels of the marker neuron-specific enolase (NSE) in lysates from AD and non-demented control brains and found no significant difference (Fig. 7G–H).

Tissue protein measurements are very sensitive to the extraction method used. To rule out extraction artifacts, we extracted a different set of human gray matter tissue (AD N = 10, age 77.9±7.7 years, MMSE 4.9±5.4/Ctrl N = 10, age 77.0±8.2 years, MMSE 29.3±1.0) with sequential extraction buffers yielding a cytosolic fraction (RAB buffer) and a membrane bound fraction (RIPA buffer). BECN1, PIK3C3, and ATG5 were predominantly found in the membranous protein fraction with BECN1 and PIK3C3 again significantly reduced in AD brain tissue and ATG5 levels unchanged (Table S1, p = 0.003 and p = 0.019).

## Discussion

Recent advances in our understanding of intracellular protein trafficking have shown that subtle alterations in the intracellular distribution of APP can have strong effects on whether it is processed by the non-amyloidogenic or amyloidogenic pathways [77]. In the current study we present data showing that autophagy is a degradative pathway that has the capacity to reduce cellular levels of APP and its metabolites when activated either physiologically (starvation), through pharmacological treatment (rapamycin or thapsigargin), or by lentiviral overproduction of Becn1. Conversely, reduced expression of Becn1 or pharmacological inhibition of autophagosomal degradation (bafilomycin A1, chloroquine, ammonium-chloride/leupeptin) led to an increase in APP and its metabolites. We conclude that Becn1 is a key regulator of cellular APP turnover.

Autophagy is a physiological mechanism that can have both beneficial and detrimental effects on neurons, depending on the circumstances [40]. Whether or not autophagy is increased in AD and whether such an increase reflects a protective attempt by cells to possibly degrade APP and Aβ, or a neurotoxic process promoting autophagic cell death has been debated. However, recent publications indicate that pharmacological stimulation of autophagy can be beneficial and reduce Aβ mediated toxicity [44], [45], [46]. In human brains and AD mouse models autophagosomes can be readily detected by electron microscopy and they appear to accumulate in swollen dystrophic neurites [7], [10], [72], [73]. This is most commonly interpreted as a sign of impaired autophagosomal degradation [7]. Furthermore APP-cleaving secretases and Aβ have been localized to autophagosomes and the accumulation of autophagosomes in AD brains and APP/PS1 mice has been interpreted as evidence that autophagy could promote AD pathology [10]. In agreement with these neuropathological findings, we observed that APP transgenic mice accumulate lysosomal and autophagosomal vesicles and that Becn1 deficiency in APP mice further promotes this pathology [41]. In addition, we confirm here that autophagy is activated in AD by detecting increased levels of LC3-II in AD brains (Fig. 7E).

**Figure 8:**
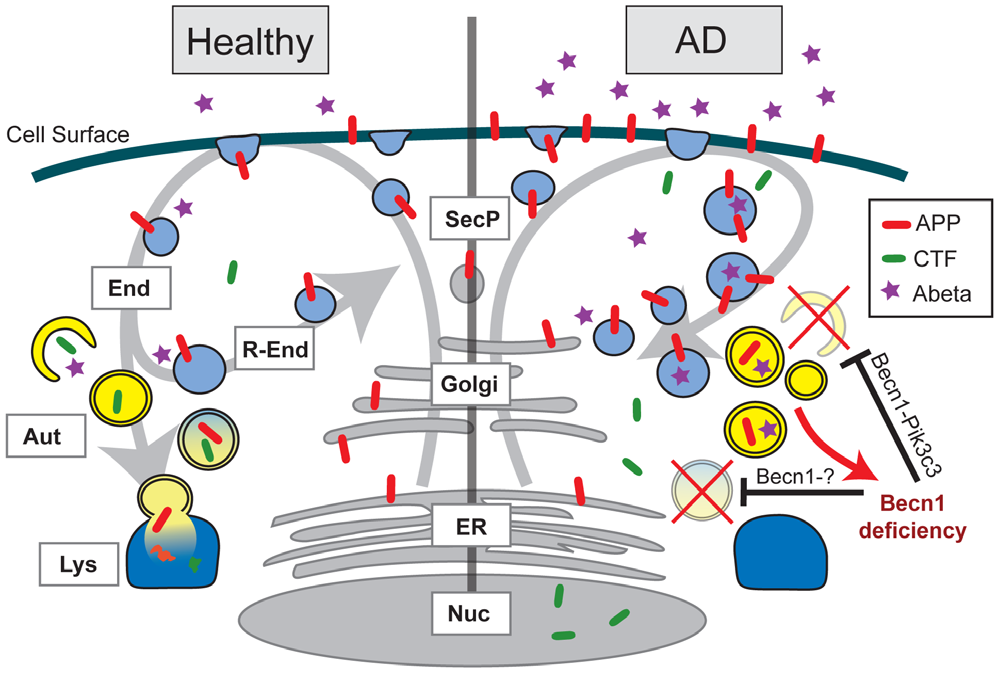
Effects of BECN1 deficiency in AD. In healthy individuals, APP is transcribed in the endoplasmatic reticulum (ER, grey), modified in the golgi network (Golgi, grey) and then shuttled to the cell surface through the secretory pathway (SecP, grey). The cell takes up APP through endocytosis (End, light blue). From here, APP can either be degraded via autophagy (Aut, yellow) and the lysosomes (Lys, dark blue) or APP can be recycled via the recycling endosomes (R-End, light blue) and enter the cycle again. In AD brains and Becn1 deficient cells BECN1 deficiency impairs both induction of autophagy (through the complex with PIK3C3) and autophagosomal degradation (potentially through a complex with an unknown binding partner). APP containing vesicles (endosomes, autophagosomes, and others) build up inside the cell. APP is increasingly cleaved by secretases and large amounts of APP-CTF and Aβ are being released, causing neurotoxic events. The disruption of autophagosomal degradation includes an increasing accumulation of autophagosomes. This accumulation can further inhibit autophagy and BECN1 expression (red arrow), worsening the reduction in APP turnover and degradation.

However, at the same time, we and others found BECN1 [41], [42], [43] and in the current study PIK3C3, reduced in AD tissue (Fig. 7B–C and Table S1), suggesting an impairment in the initiation of autophagy. To reconcile these apparently contradictory findings we postulate a dual role for BECN1: one in autophagy initiation, in a complex with PIK3C3, and another in autophagosomal flux and degradation, potentially in a complex with other proteins (Fig. 8). BECN1 has been shown to execute various functions depending on its binding partners and siRNA mediated knockdown of *Becn1* has been demonstrated to impair autophagosomal degradation and cause LC3-II accumulation at the same time [57], similar to our findings (Fig. 4A&C). Different experimental models therefore appear to reflect different aspects of this dual role. On one hand *Becn1* heterozygous knockout mice have reduced autophagosomes and reduced LC3-II [41], reflecting impaired autophagosomal initiation. On the other hand, *Becn1* siRNA treated CHO cells have increased LC3-II levels (Fig. 4A&C). This reflects impaired autophagosomal degradation similar to the pathology observed in AD brains. In either role, reducing BECN1 leads to pathological accumulations of APP and its metabolites through impaired autophagy (Fig. 8).

Our observations regarding the effect of *Becn1* siRNA on autophagy confirm that the BECN1-PI3K complex has a crucial role during the initiation stages of autophagy, but they also show that reduction of Becn1 protein levels can have effects on the availability of PIK3C3 and vice versa (Fig. 4D–G). This is supported by recent findings of similar Becn1 reduction after *Pik3c3* knockdown [78], although a reduction of Pik3c3 after *Becn1* knockdown had not been reported. It will be important do determine if other proteins that are part of the BECN1 complex (Atg14L, PIK3R4, UVRAG, Ambra1, Vps15, Bif-1, or Rubicon) are also reduced in AD or in response to BECN1 reduction, respectively, as this could help explain the (possibly indirect) effects of BECN1 reduction on autophagosomal degradation (Fig. 8). Atg14L and UVRAG are especially interesting candidates for this since both proteins have been shown to determine the stability of Becn1 [78] and Atg14L knockdown causes LC3-II accumulation similar to Becn1 siRNA [57]. Further studies will be needed to precisely determine the role of Becn1 and its binding partners in the modulation of autophagic flux and autophagosomal maturation. Nevertheless, with respect to APP metabolism, Becn1 seems to play central role, since Pik3c3 siRNA does not cause a comparable effect on APP accumulation in our *in vitro* system (Fig. 4F–G).

Aiming to validate our cell culture findings in AD brain tissue, we measured the levels of PIK3C3, LC3, and ATG5. We found a reduction not only of BECN1, but also of its binding partner PIK3C3, similar to our cell culture model using *Becn1* siRNA (Fig. 7C). Importantly, we observed a linear relationship between the levels of these two proteins (Fig. 7D) similar to the cell culture studies, supporting the idea that reduction in one of the proteins can cause instability of the PI3K complex and increased degradation or reduced production of the respective binding partner. The levels of ATG5 on the other hand were not significantly changed, arguing for a specific disruption of the PI3K complex in AD rather than a general deficiency in the autophagy pathway and signaling cascade. The reduction in PI3K complex components appear to have an inhibiting effect on the degradation rate of autophagosomes, which may lead to the build-up of LC3 protein in brain tissue and a subsequent accumulation of APP and its metabolites.

Which comes first, BECN1/PIK3C3 deficiency or APP accumulation? While the data from the transgenic mice suggested an important role of Becn1 levels on AD pathology [41], it was unclear if this effect is upstream of APP pathology or partially a consequence of disrupted intracellular trafficking due to APP overexpression. Our cell culture data from wildtype human APP overexpressing cell lines demonstrate now that APP overexpression alone does not lead to reduced Becn1 and Pik3c3 levels, leaving the possibility that autophagy disturbance could precede APP/Aβ pathology *in vivo*, and that the observed reduction of BECN1 in human AD brain tissue is unlikely due to elevated levels of APP or its metabolites alone. Instead, it suggest that an escalating disturbance in autophagosomal flux and degradation could have a negative impact on BECN1 and/or PIK3C3 levels, presumably via a negative feedback loop, downregulating autophagy induction in response to abundant autophagosome numbers (Fig. 8). Such a loop could be in place to prevent an uncontrolled run-off activation of autophagy with potentially disastrous consequences for the cell. In support of such a model, pharmacological inhibition of autophagosomal-lysosomal fusion using BafA causes a strong accumulation of autophagosomes, accompanied with APP and APP-CTF accumulation in those autophagosomes and other intracellular vesicles. This in turn leads to decreased levels of Becn1 and, at least under some treatment conditions, of Pik3c3 (Fig. 5L). These findings suggest that disturbances in autophagosome turnover can further inhibit proper induction and execution of autophagy, potentially worsening the cellular capacity to degrade APP and its metabolites.

The initial factor that impairs autophagy in AD and reduces BECN1/PIK3C3 still has to be determined. This study however identifies autophagy as an important degradative pathway for APP and suggests that once autophagosomal flux and turnover is impaired an escalating cycle of APP/APP-CTF/Aβ accumulation and further reduced initiation of autophagy occurs (Fig. 8). Future studies of conditional knockout mice for proteins that are part of the BECN1-PI3K complex will help to deepen our understanding of the sequence of events that lead to the disruption of autophagy and how this contributes to the development of AD pathology.

## Materials and Methods

### Cell culture

B103/hAPPwt rat neuroblastoma cells were maintained in Dulbecco's modified Eagle's medium (Invitrogen, Carlsbad/CA, USA) containing 10% (v/v) fetal bovine serum and 5% (v/v) horse serum at 37°C with 5% CO_2_. Selection was maintained with 400 µg/ml geneticin/G418 (Invitrogen). CHO/hAPPwt, APLP1 and APLP2 hamster ovary cells were maintained in Dulbecco's modified Eagle's medium containing 10% (v/v) fetal bovine serum and selection maintained using 500 µg/ml hygromycin (Invitrogen).

### Drug treatments/Starvation

Cells were washed once in warm PBS and covered with fresh medium containing drugs at the indicated concentrations/for the indicated periods: 100 nM rapamycin for 90 min (Calbiochem, San Diego/CA, USA); 3 µM/1 µM thapsigargin for 12 hrs (Calbiochem, San Diego/CA, USA); 50 nM/100 nM bafilomycin A1 for 24 hrs (LC Laboratories, Woburn/MA, USA); 20 mM ammoniumchloride and 10 µg/ml leupeptin (Sigma-Aldrich, St. Louis/MO, USA) for 24 hrs; 30 µg/ml chloroquine (Sigma-Aldrich, St. Louis/MO, USA) for 16 hrs; 100 nM DAPT (Sigma-Aldrich, St. Louis/MO, USA) for 24 hrs. Control cells were treated with the corresponding amount of vehicle. At the end of the incubation period the cells were harvested or imaged as described below. For starvation experiments, the cells were washed twice in warm PBS and then incubated for 90 min in HANKS or 4 hrs in DPBS (Invitrogen, Carlsbad/CA, USA) solution.

### Antibodies

The following primary antibodies were used: BECN1 antibody #612112 1∶500 (BD Biosciences, San Jose/CA, USA); LC3 antibody #PD014 1∶500 WB/1∶200 IHC (MBL International, Woburn/MA, USA); PIK3C3 antibody #38-2100 1∶500 (Zymed, San Francisco/CA, USA); Actin antibody #A-5060 1∶10000 (Sigma-Aldrich, St. Louis/MO, USA); Atg5 antibody 1∶2000 (gift from Dr. Noburo Mizushima, Tokyo Metropolitan Institute of Medical Science, Japan); N-terminal APP 8E5 antibody 1∶5000(WB)/1∶200(IHC) (gift from Elan, South San Francisco/CA, USA); C-terminal APP CT15/CT20 antibody 1∶1000(WB)/1∶200(IHC) (gift from Dr. Todd Golde, Mayo Clinic, Jacksonville/FL, USA); APLP1 antibody #171615 1∶5000 (Calbiochem, SanDiego/CA, USA); APLP2 antibody #171616 1∶5000 (Calbiochem, SanDiego/CA, USA); NSE antibody #MS-171-P1 1∶1000 (LabVision, Fremont/C, USA).

### RNAi and LV particles

B103/hAPPwt, CHO/hAPP, CHO/hAPLP1 or CHO/hAPLP2 cells were transfected with 40 nM synthetic Stealth siRNA (Invitrogen) using Lipofectamine 2000 (Invitrogen, Carlsbad/CA, USA) following manufacturers instructions. The siRNA sequences used were as follows:

BECN1: CCCAGCCAGGAUGAUGUCUACAGAA and GCUAACUCAGGAGAGGAGCCAUUUA.

PIK3C3: CAUUGCCGUUAGAGCCACAGGUGAA and GGAGCCUACCAAGAAGGAUAGUCAA.

Control: GCUACUCGAGGAGGAACCGUAAUUA.

For LV experiments the cells were transduced with virus containing a shRNA plasmid against mBecn1 targeting the nucleotides 405–423 (or against mAtg5) and a GFP-marker. The control LV contained the empty plasmid with only the GFP-marker. For the Becn1 overexpression experiments, the LV particles contained a plasmid encoding mBecn1 alone. Cells were transduced in 96 well plates at 50 MOI in the presence of polybrenen (8 µg/ml). Successful transduction was monitored by GFP expression. Following the transduction and expansion the cells were stained or lysed after 36–96 hrs. All LV particles were provided by Dr. E. Masliah, University of California San Diego/CA, USA.

### Protein extraction

Samples from human brain tissue were homogenized in extraction buffer (see blow) by pulsed ultrasonification at 4°C, followed by centrifugation at 10000×g at 4°C for 30 min. The resulting supernatant was used for protein analysis. For cell culture samples, cells were washed once with PBS (Invitrogen, Carlsbad/CA, USA) and scraped off the plate. After a brief centrifugation at 4500×g at 4°C for 5 min, the cell pellets were re-suspended in extraction buffer and homogenized by pipetting, three freeze-thaw cycles on dry ice, and 30 min incubation on ice. Insoluble particles were pelleted by centrifugation with 10000×g at 4°C and the resulting supernatant was used for analysis. Proteins were extracted using RIPA buffer (50 mM HCl, 150 mM NaCl, 5 mM EDTA, 1 mM EGTA, 20 mM NaF, 1 mM Na_2_VO_4_, 1% NP40, 0.5% Sodium deoxycholate, 1 mM PMSF, 0.1% SDS, pH 7.4) containing 2x Complete proteinase inhibitor (Roche Diagnostics, Mannheim, Germany). When sequential extraction was performed the samples were first extracted with detergent free RAB buffer (MES 100 mM, EGTA 1 mM, MgSO_4_ 0.5 mM, NaCl 750 mM, NaF 20 mM, EDTA 100 mM, Na_2_VO_4_ 1 mM, PMSF 1 mM, pH 6.5) containing 2x Complete proteinase inhibitor (Roche Diagnostics, Mannheim, Germany) followed by a RIPA extraction of the pellet.

### Western blotting

A pre-cast bis-tris gel (Invitrogen, Carlsbad/CA, USA) and a MOPS buffer system were used and standard Western blotting protocols were followed. 10–20 µg of total protein were loaded. Gels were transferred onto 0.4 µm nitro-cellulose membranes (BioRad, Hercules/CA, USA) and pre-incubated with MISER antibody extender solution (Pierce, Rockford/IL, USA). Total protein was measured with the BCA Protein Assay Kit (Thermo Scientific, Rockford/IL, USA) against a BSA standard. Antigen specific primary antibodies were incubated 1 hr at room temperature or overnight at 4°C and detected with species-specific horseradish-peroxidase coupled secondary antibodies. The ECL Western Blotting Detection kit (GE Healthcare, Buckinghamshire, UK) was used to obtain a chemiluminescence signal, which was then detected using Amersham Hyperfilm ECL (GE Healthcare, Buckinghamshire, UK) at varying exposure times to obtain images with optimal density within the dynamic range of the film (30 s–30 min). The films were digitalized at 300dpi and arranged in Photoshop CS4 (Adobe, San Jose/CA, USA) as TIFF files. Band quantification was performed using ImageJ software (NIH, Bethesda/MD, USA). Bands of interest were normalized to a loading control using Microsoft Excel 2008 (Microsoft Corporation, Seattle/WA, USA) and statistical analysis and graph production was performed in Prism5 (GraphPad Software, La Jolla/CA, USA).

### A*β* ELISA

ELISAs were performed as described [41] using antibody 266 (Aβ_13–28_, Elan) as the capture antibody for total Aβ, or antibody 21F12 (Aβ_37–42_, Elan, South San Francisco/CA, USA) as the capture antibody for Aβ_x-42_ and biotinylated 3D6 (Aβ_1–5_, Elan, South San Francisco/CA, USA) as the detection antibody. After incubation with the secondary antibody, samples were incubated with avidin-HRP and the signal developed using “1-step slow TMB ELISA solution” (Thermo Scientific, Rockford/IL, USA). For the thapsigargin-treatment experiments, we used a MesoScale detection system (Gaithersburg/MD, USA) and followed the standard protocol with the above antibodies.

### Fluorescence Microscopy

For epifluorescence microscopy cells were grown in 12 well tissue culture plates (Becton Dickinson Labware, Franklin Lakes/NJ, USA). They were washed with ice-cold PBS and then fixed in cold 4% PFA in phosphate buffer for 5 min at 4°C followed by 10 min at RT. Cells were then washed three times with ice-cold PBS and PFA fluorescence was quenched with ice-cold 100 mM tris-HCl pH 8.0 for 3 min. The cells were then either washed three times in ice-cold PBS and stained (for cell surface APP) or permeabilized with ice-cold methanol for 6 min at −20°C, followed by three washes of ice-cold PBS and staining (for intracellular proteins). Staining was performed by blocking cells in blocking buffer (4% donkey serum, 2% bovine serum albumin, 2% fetal calf serum, 0.2% fish gelatin in PBS) for 1 hr at RT. Primary antibodies in blocking buffer were applied to the cells for 1 hr at RT, followed by three 5 min washes in PBS. Fluorescent secondary antibodies in blocking buffer were added and incubated for 1 hr at RT, followed by three washes in PBS for 5 min. Cells were visualized with a Olympus IX71 microscope with a CoolSnapHQ camera (Roper Scientific, Tucson/AZ, USA). Image analysis was done with MetaMorph 6.1r6 (Molecular Devices, Sunnyvale/CA, USA). For confocal microscopy cells were grown on glass cover slips (Fisher Scientific, Hampton/NH, USA) in 12 well plates, and fixed and stained similar to the epifluorescence protocol above. The glass coverslips were mounted in MoWiol and visualized on a Zeiss LSM 510 confocal microscope. Image analysis was done with the Zeiss LSM software package.

### RT-PCR

RNA was extracted from B103/hAPP cells (n = 5 wells per treatment group) using Trizol and cleaned using RNAeasy mini kit (Qiagen, Valencia/CA, USA). cDNA was synthesized using TaqMan reverse transcriptase (Applied Biosystems, Branchburg/NJ, USA). cDNA was amplified in triplicate on a MyiQ single color real time PCR detection system using primers specific to human APP (F 5′CACCAATGTGGTAGAAGCCAACC3′, R 5′GGGCAACACACAAACTCTACCCC3′), and GAPDH (F 5′TGCGACTTCAACAGCAACTC3′, R 5′ATGTAGGCCATGAGGTCCAC3′). The PCR cycle was as follows: 10 min at 95°C, 45 x (30 s at 95°C, 2 min at 60°C, 30 s at 72°C). Cycle numbers for amplification to exceed a pre-set threshold were used to determine the APP mRNA copy number. cDNA prepared without reverse transcriptase was amplified to ensure no genomic DNA contamination of the samples.

### Human brain tissue

Brain tissues from confirmed AD and age-matched, non-demented, non-pathological controls were obtained from ADRC at the University of California - San Diego, The Institute for Brain Aging and Dementia Tissue Repository at the University of California - Irvine, and Stanford Brain Bank at Stanford University in strict accordance with all ethical and institutional guidelines. Cortical mid-frontal gray matter tissue was cut out of frozen tissue blocks and subject to protein extraction as described above.

### Statistics

Human brain tissue protein data consists of one-sample measurements for each case. The data was normalized against actin and differences calculated using Student's unpaired t-test. Cell culture western blots experiments were conducted in two to three independent experiments consisting of duplicates or triplicates. All measurements were normalized by actin intensities and then calculated as levels relative to control conditions. Differences between treatment conditions were established using student's unpaired t-test (with two conditions) or one-way ANOVA followed by Dunnett's test for multiple comparisons (for more than two conditions). For fluorescence microscopy, stains were done in independent duplicates and representative images chosen.

## Supporting Information

Figure S1 Expression of Becn1 and Pik3c3 in the mouse brain, especially in the hippocampus, indicates an important function of autophagy in neuronal homeostasis (from the Allen Mouse Brain Atlas. Seattle (WA): Allen Institute for Brain Science. Available from http://mouse.brain-map.org).
(0.38 MB TIF)

Figure S2 Control or Atg5 shLV treated B103/hAPP cells were starved in DPBS for 4 hours. Atg5 and APP levels were measured by Western-blotting and quantified. Atg5 reduction significantly impairs starvation induced autophagosomal APP degradation (Data is from the same blot. The vertical line indicates removal of three lanes not part of this experiment.) Bars are mean ± SEM from triplicate cultures. * p<0.05, ** p<0.01, *** p<0.001 by unpaired Student's t test.
(0.16 MB TIF)

Figure S3 Quantification of B103/hAPP RIPA cell lysates, 72 hours after siRNA kockdown. All bars are mean ± SEM. Means from at least two independent experiments were compared by unpaired Student's t test. * p<0.05, ** p<0.01, *** p<0.001
(0.14 MB TIF)

Figure S4 Epifluorescence microscopy of CHO/hAPP cells treated with Becn1 siRNA for 48 hours. All bars are mean ± SEM. Means from at least two independent experiments were compared by unpaired Student's t test. * p<0.05, ** p<0.01, *** p<0.001
(0.85 MB TIF)

Figure S5 Western-blot of control or Becn1 shLV transduced B103/hAPP cells that were treated with vehicle or 100 nM DAPT for 24 hours. An anti-luciferase shLV was used as control.
(0.30 MB TIF)

Figure S6 Western-blots and quantification of CHO/hAPP and B103/hAPP cells treated with chloroquine (CQ) or ammoniumchloride/leupeptin (NL). Means from three independent experiments were compared by unpaired Student's t test. * p<0.05, ** p<0.01, *** p<0.001
(0.45 MB TIF)

Figure S7 A–B. Control experiments for the LV overexpression of Becn1. Control for cell size as a measure of physiological cell health (A). High Becn1 overexpressors exhibit either swollen or shrunken cell bodies, indicating non-physiological stress. Quantification (B) of APP, Becn1 immunofluorescence, and cell size in GFP LV control cells (N = 100) shows no difference in APP or Becn1 levels for low and medium overexpression of GFP. High GFP expression induces non-physiological conditions leading to an unspecific accumulation of Becn1 and APP and cell shrinkage. This led us to not further explore the effects of the highest Becn1 or GFP expressing cells.
(0.19 MB TIF)

Table S1 Human cortical gray matter tissue was subject to sequential RAB/RIPA buffer extraction and Western blotting. Control (N = 10) and AD (N = 10) cases were compared regarding their relative BECN1, PIK3C3, and ATG5 levels. While BECN1 and PIK3C3 levels were significantly reduced in AD brains when compared to controls, no difference was detectable in ATG5 levels.
(0.03 MB DOC)
