# Supplementary figures and images for: Regulation of Amyloid Precursor Protein Processing by the Beclin 1 Complex
Source: PLoS One. 2010 Jun 15;5(6):e11102. doi: 10.1371/journal.pone.0011102 (PMC2886067; doi:10.1371/journal.pone.0011102)

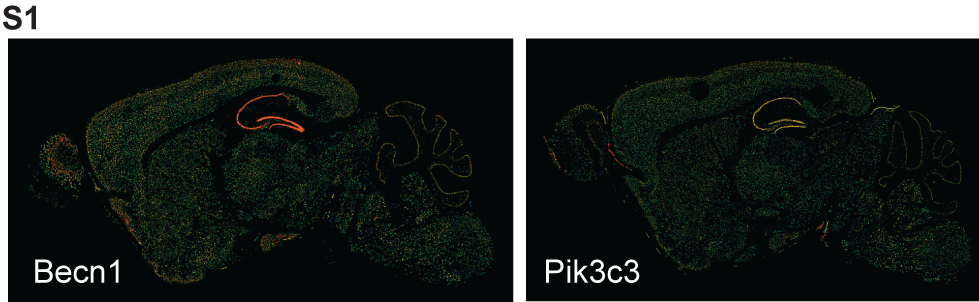

Supplement: Figure S1 — Expression of Becn1 and Pik3c3 in the mouse brain, especially in the hippocampus, indicates an important function of autophagy in neuronal homeostasis (from the Allen Mouse Brain Atlas. Seattle (WA): Allen Institute for Brain Science. Available from http://mouse.brain-map.org). (0.38 MB TIF) [file pone.0011102.s001.tif]

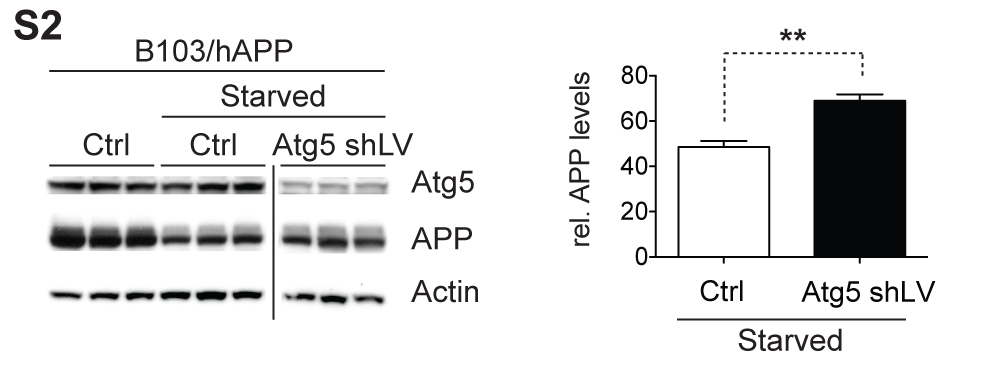

Supplement: Figure S2 — Control or Atg5 shLV treated B103/hAPP cells were starved in DPBS for 4 hours. Atg5 and APP levels were measured by Western-blotting and quantified. Atg5 reduction significantly impairs starvation induced autophagosomal APP degradation (Data is from the same blot. The vertical line indicates removal of three lanes not part of this experiment.) Bars are mean ± SEM from triplicate cultures. * p<0.05, ** p<0.01, *** p<0.001 by unpaired Student's t test. (0.16 MB TIF) [file pone.0011102.s002.tif]

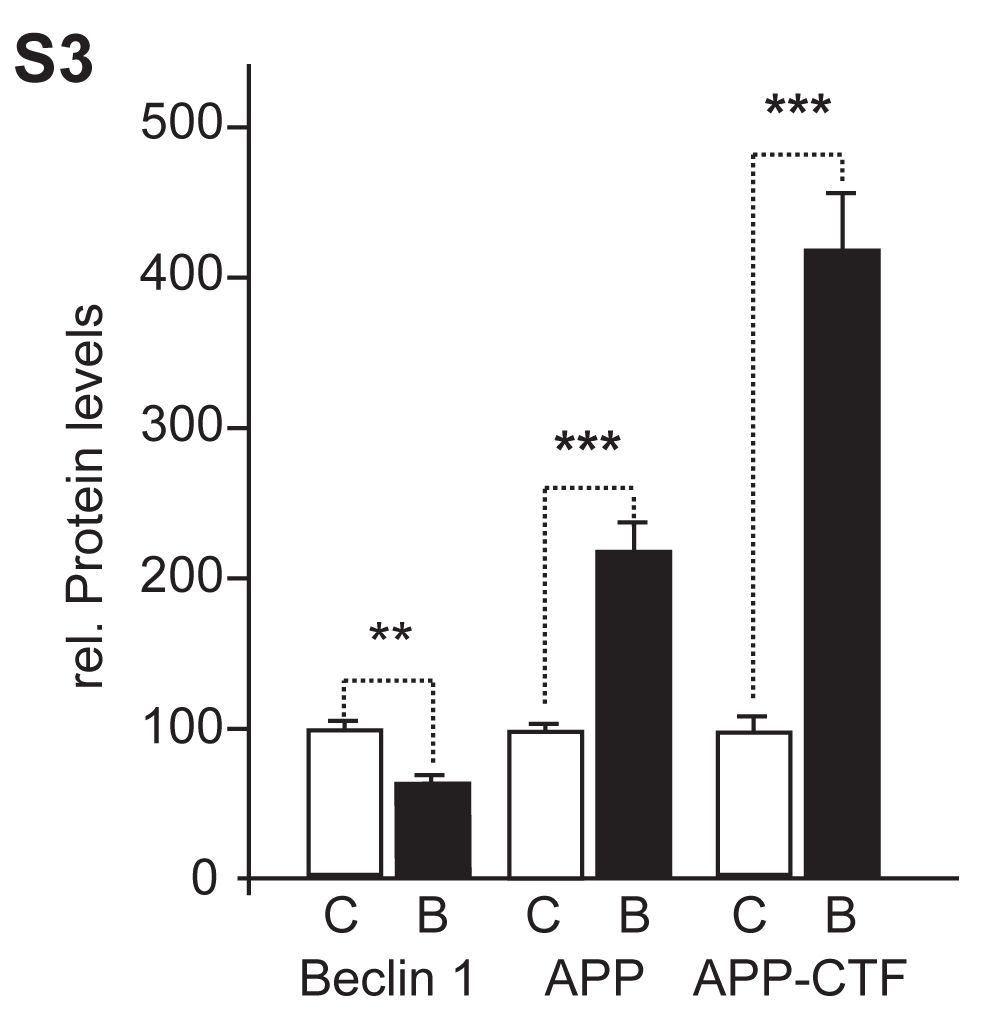

Supplement: Figure S3 — Quantification of B103/hAPP RIPA cell lysates, 72 hours after siRNA kockdown. All bars are mean ± SEM. Means from at least two independent experiments were compared by unpaired Student's t test. * p<0.05, ** p<0.01, *** p<0.001 (0.14 MB TIF) [file pone.0011102.s003.tif]

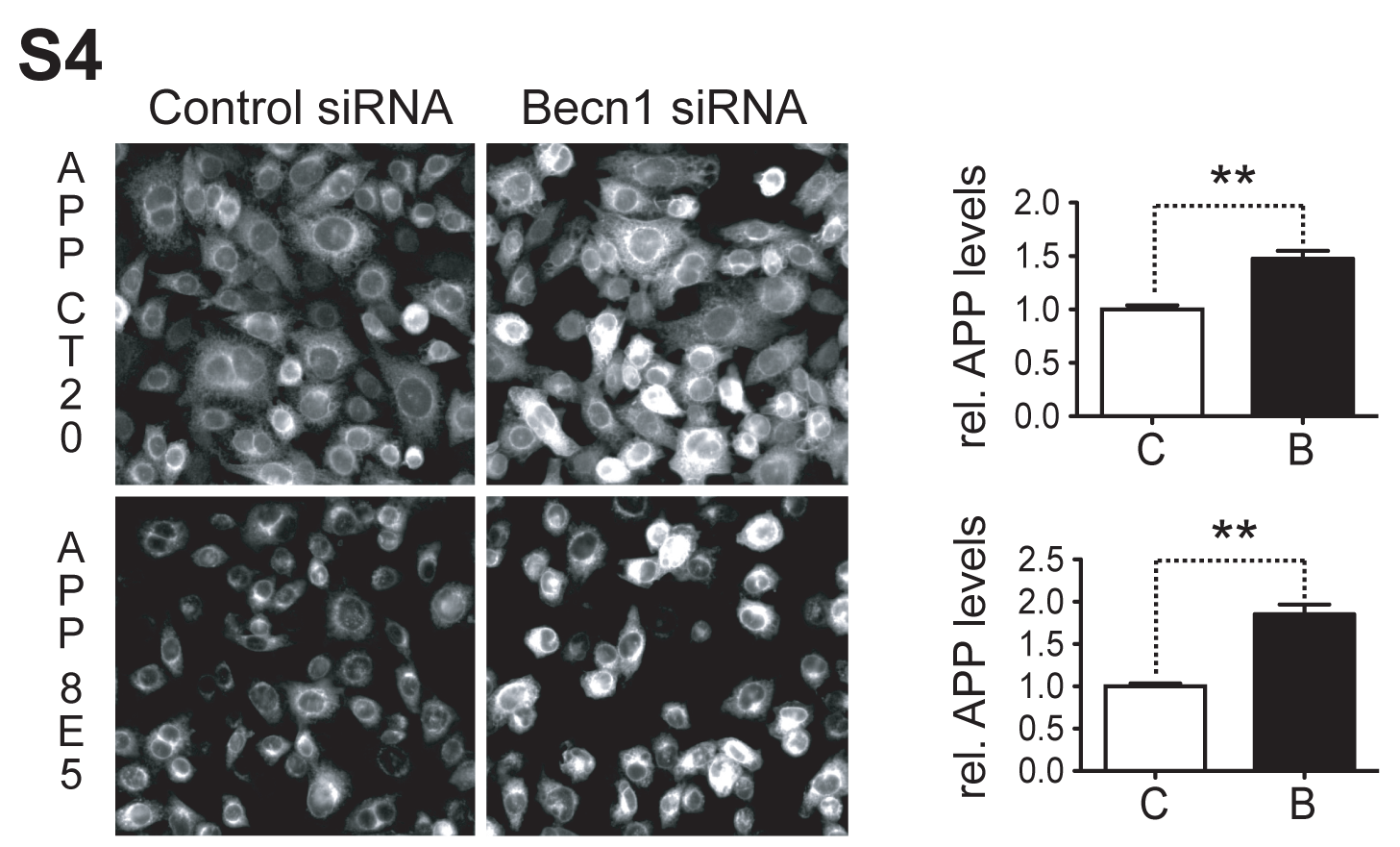

Supplement: Figure S4 — Epifluorescence microscopy of CHO/hAPP cells treated with Becn1 siRNA for 48 hours. All bars are mean ± SEM. Means from at least two independent experiments were compared by unpaired Student's t test. * p<0.05, ** p<0.01, *** p<0.001 (0.85 MB TIF) [file pone.0011102.s004.tif]

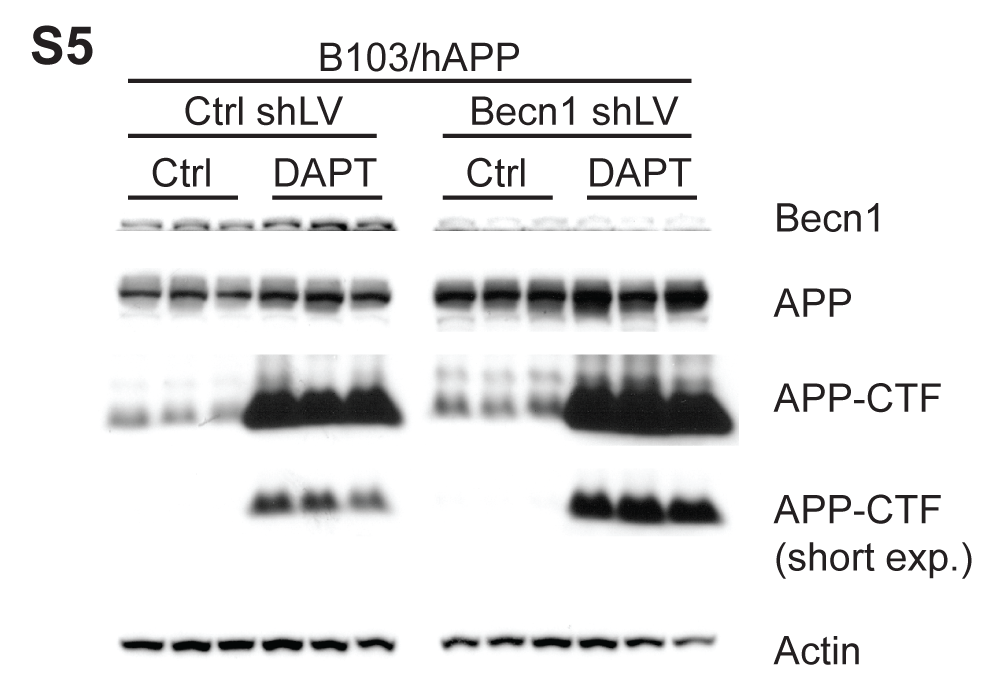

Supplement: Figure S5 — Western-blot of control or Becn1 shLV transduced B103/hAPP cells that were treated with vehicle or 100 nM DAPT for 24 hours. An anti-luciferase shLV was used as control. (0.30 MB TIF) [file pone.0011102.s005.tif]

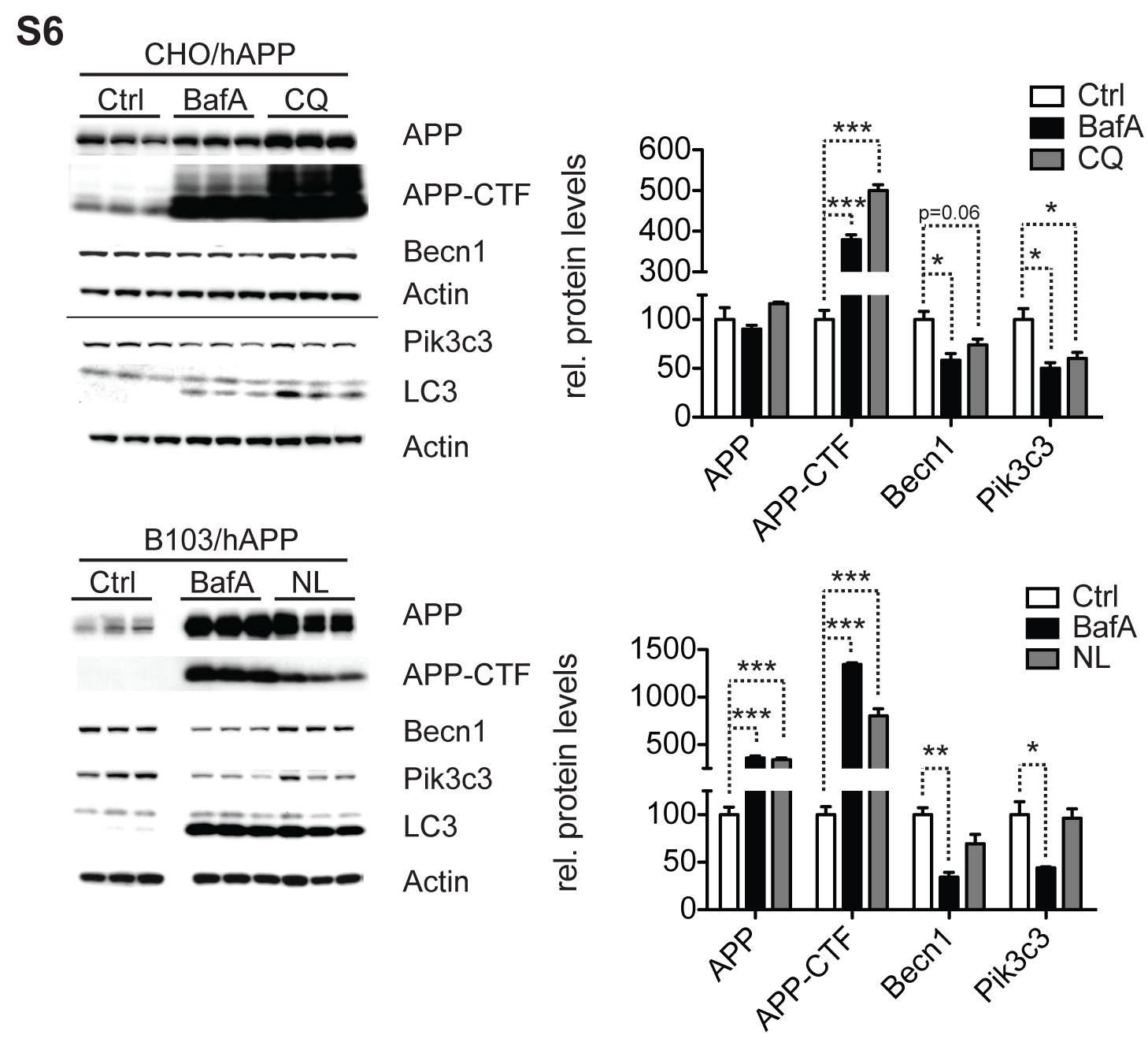

Supplement: Figure S6 — Western-blots and quantification of CHO/hAPP and B103/hAPP cells treated with chloroquine (CQ) or ammoniumchloride/leupeptin (NL). Means from three independent experiments were compared by unpaired Student's t test. * p<0.05, ** p<0.01, *** p<0.001 (0.45 MB TIF) [file pone.0011102.s006.tif]

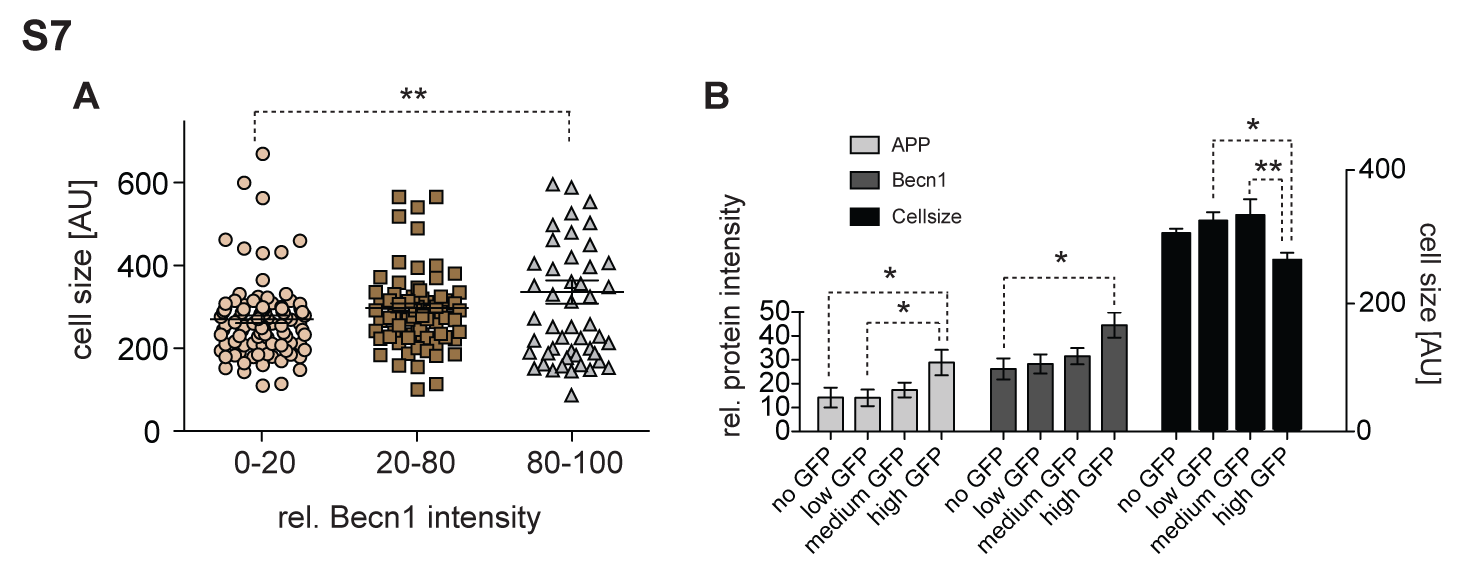

Supplement: Figure S7 — A–B. Control experiments for the LV overexpression of Becn1. Control for cell size as a measure of physiological cell health (A). High Becn1 overexpressors exhibit either swollen or shrunken cell bodies, indicating non-physiological stress. Quantification (B) of APP, Becn1 immunofluorescence, and cell size in GFP LV control cells (N = 100) shows no difference in APP or Becn1 levels for low and medium overexpression of GFP. High GFP expression induces non-physiological conditions leading to an unspecific accumulation of Becn1 and APP and cell shrinkage. This led us to not further explore the effects of the highest Becn1 or GFP expressing cells. (0.19 MB TIF) [file pone.0011102.s007.tif]
