# Supplementary material for: Regulation of Amyloid Precursor Protein Processing by the Beclin 1 Complex
Source: PLoS One. 2010 Jun 15;5(6):e11102. doi: 10.1371/journal.pone.0011102 (PMC2886067; doi:10.1371/journal.pone.0011102)
Supplement: Table S1 — Human cortical gray matter tissue was subject to sequential RAB/RIPA buffer extraction and Western blotting. Control (N = 10) and AD (N = 10) cases were compared regarding their relative BECN1, PIK3C3, and ATG5 levels. While BECN1 and PIK3C3 levels were significantly reduced in AD brains when compared to controls, no difference was detectable in ATG5 levels. (0.03 MB DOC) [file pone.0011102.s008.doc]

|  | | **Cytosolic (RAB)** | | | **Membranous (RIPA)** | | |
| --- | --- | --- | --- | --- | --- | --- | --- |
| **Protein** | **Disease** | **Mean** | **Stdev** | **p value** | **Mean** | **Stdev** | **p value** |
| BECN1 | Control  AD | n.d. |  |  | 0.51  0.30 | 0.05  0.03 | 0.003  ** |
| PIK3C3 | Control  AD | n.d. |  |  | 0.76  0.41 | 0.10  0.07 | 0.019  * |
| ATG5 | Control  AD | 0.37  0.19 | 0.11  0.03 | 0.168 | 1.79  2.10 | 0.14  0.19 | 0.20 |

**Supplementary Table 1**
